# β3-adREnoceptor Analysis in CORD Blood of Neonates (β3 RECORD): Study Protocol of a Pilot Clinical Investigation

**DOI:** 10.3390/life14060776

**Published:** 2024-06-19

**Authors:** Rosa Teresa Scaramuzzo, Stefania Crucitta, Marzia del Re, Maurizio Cammalleri, Paola Bagnoli, Massimo Dal Monte, Alessandro Pini, Luca Filippi

**Affiliations:** 1 Neonatology Unit, Azienda Ospedaliero Universitaria Pisana, 56126 Pisa, Italy; 2 Clinical Pharmacology and Pharmacogenetics Unit, Department of Clinical and Experimental Medicine, University of Pisa, 56126 Pisa, Italy; stefania.crucitta@unipi.it (S.C.); marzia.delre@unipi.it (M.d.R.); 3 Unit of General Physiology, Department of Biology, University of Pisa, 56126 Pisa, Italy; maurizio.cammalleri@unipi.it (M.C.); paola.bagnoli@unipi.it (P.B.); massimo.dalmonte@unipi.it (M.D.M.); 4 Department of Experimental and Clinical Medicine, University of Florence, 50121 Florence, Italy; alessandro.pini@unifi.it; 5 Neonatology Unit, Department of Clinical and Experimental Medicine, University of Pisa, 56126 Pisa, Italy

**Keywords:** prematurity, preterm newborn, oxygen, vascularization

## Abstract

**Background and Objective**: The embryo and the fetus develop in a physiologically hypoxic environment, where vascularization is sustained by HIF-1, VEGF, and the β-adrenergic system. In animals, β3-adrenoceptors (β3-ARs), up-regulated by hypoxia, favor global fetal wellness to such an extent that most diseases related to prematurity are hypothesized to be induced or aggravated by a precocious β3-AR down-regulation, due to premature exposure to a relatively hyperoxic environment. In animals, β3-AR pharmacological agonism is currently investigated as a possible new therapeutic opportunity to counteract oxygen-induced damages. Our goal is to translate the knowledge acquired in animals to humans. Recently, we have demonstrated that fetuses become progressively more hypoxemic from mid-gestation to near-term, but starting from the 33rd–34th week, oxygenation progressively increases until birth. The present paper aims to describe a clinical research protocol, evaluating whether the expression level of HIF-1, β3-ARs, and VEGF is modulated by oxygen during intrauterine and postnatal life, in a similar way to animals. **Materials and Methods**: In a prospective, non-profit, single-center observational study we will enroll 100 preterm (group A) and 100 full-term newborns (group B). We will collect cord blood samples (T0) and measure the RNA expression level of HIF-1, β3-ARs, and VEGF by digital PCR. In preterms, we will also measure gene expression at 48–72h (T1), 14 days (T2), and 30 days (T3) of life and at 40 ± 3 weeks of post-menstrual age (T4), regardless of the day of life. We will compare group A (T0) vs. group B (T0) and identify any correlations between the values obtained from serial samples in group A and the clinical data of the patients. Our protocol has been approved by the Pediatric Ethical Committee for Clinical Research of the Tuscany region (number 291/2022). **Expected Results**: The observation that in infants, the HIF-1/β3-ARs/VEGF axis shows similar modulation to that of animals could suggest that β3-ARs also promote fetal well-being in humans.

## 1. Introduction

It is well known that during intrauterine life, embryonic and fetal well-being is guaranteed by low levels of oxygen [1], which ensure the growth and vascularization of both the embryo and the fetus [2,3]. The biological mechanisms of this phenomenon are only partially known, and most of our knowledge comes from studies performed in animal models. A more in-depth knowledge of these mechanisms, preferentially in humans, would represent notable progress, firstly to better understand (and possibly favor) fetal well-being, but also to better understand the mechanisms involved in the development of diseases related to premature birth.

While the role of hypoxia and hypoxia-related genes, i.e., Hypoxia-Inducible Factor-1 (HIF-1) and some of its target genes, including the Vascular Endothelial Growth Factor (VEGF), in ensuring the well-being and growth of the embryo and the fetus have been known for many years, the function of the adrenergic system, and specifically that of the β3-adrenoreceptor (β3-AR), is the subject of recent research. β3-AR is the focus of our translational research, aimed to verify whether in humans, the behaviors and functions of this receptor is similar to those recently revealed in animals.

### 1.1. Background

The transcription factor HIF-1 is the master regulator of responses to hypoxia. Indeed, in hypoxic conditions HIF-1 regulates the expression of a series of angiogenic factors, such as VEGF, which is essential in promoting embryonic and fetal vascularization [2,3].

HIF-1 consists of two subunits, HIF-1α and HIF-1β, encoded by the Hif1a and Hif1b genes, respectively. Both HIF-1α and HIF-1β subunits are constitutively expressed, but only HIF-1α is affected by oxygen levels. In normoxia, HIF-1α is hydroxylated by oxygen-sensitive prolyl hydroxylase domain-containing proteins, thus recognized by the von Hippel-Lindau protein, which acts as an E3 ubiquitin ligase, and rapidly degraded by the proteasome [4,5]. In hypoxic conditions, such as during intrauterine life, its lack of hydroxylation leads to the upregulation of HIF-1: HIF-1α subunit is translocated to the nucleus to form a heterodimer with HIF-1β, which then binds to hypoxia-responsive elements (HREs) on DNA and activates the transcription of several hundred hypoxia-responsive genes, whose products participate in regulating different strategies to promote embryonic and fetal well-being and growth.

Adaptive processes, such as vascularization, erythropoiesis, and cell survival are thus triggered by hypoxia, but enzymatic systems are also activated to favor the transition from oxidative phosphorylation to glycolysis. As an effect, the up-regulation of HIF-1 during the embryonic/fetal life activates a particular metabolic shift, i.e., the so-called Warburg effect [6], which is less profitable in the production of ATP but less dependent on oxygen. The activation of this metabolic reprogramming leads to the release of large quantities of lactic acid, which is fundamental to favoring embryonic implantation, as well as to the production of pentose phosphates, which is essential to supporting intense proliferative activity [7]. In addition, HIF-1 also participates in ensuring embryonic/fetal well-being by promoting the induction of an immunotolerant [8,9] and chemoresistant environment [10,11,12].

However, the full spectrum of HIF-1 and the fine mechanisms that allow HIF-1 to perform these functions are not yet fully known. Much remains to be understood about the target genes and related pathways activated by HIF-1.

Among the numerous genes directly or indirectly regulated by HIF-1, the VEGF gene family certainly deserves mention. The most important protein in this group is VEGF-A, although the VEGF gene family also includes PlGF (placental growth factor, a protein involved in prenatal development), VEGF-B, VEGF-C, and VEGF-D. Further molecules of this family have been discovered in viruses (VEGF-E) and in the venom of some snakes (VEGF-F). The activity of VEGF-A has been mostly studied on vascular endothelial cells, although it has important repercussions on numerous other cell types (monocytes and macrophages, neurons, tumor cells, kidney epithelial cells, etc.). In vitro, it has been shown to stimulate endothelial cell mitosis and migration. It is also a vasodilator and increases vascular permeability. During embryonic and fetal development, VEGF is expressed in multiple tissues, with the highest levels found in the lungs, kidneys, and heart. VEGF is also expressed in placental tissues and fetal membranes, and this expression increases with advancing gestation [13].

The role of hypoxia, and therefore of HIF-1 and VEGF, in ensuring the well-being and growth of the embryo and fetus has been known for many years. At the same time, since the end of the 1990s, the decisive role played by the adrenergic system in ensuring fetal survival has emerged, as suggested by the high embryo lethality observed in mice lacking tyrosine hydroxylase, the rate-limiting enzyme in catecholamine biosynthesis [14,15,16]. Adrenoceptors are well-known as central mediators of the sympathetic nervous system. Binding catecholamines (adrenaline and noradrenaline), adrenoceptors exert their effects by regulating the function of organs and tissues in maintaining homeostasis and in the response to stress. The different subtypes of ARs identified so far (nine in total) are divided into three families (α1, α2, β). Early studies had demonstrated that the effect of catecholamines on fetal cardiac reactivity and fetal survival was mediated generically by their action on β-adrenoceptors (β-ARs), without evaluating which receptor was most involved [17]. However, subsequent studies identified β1-AR as the β-AR subtype most involved in maintaining fetal heart rate in a hypoxic environment [18].

β3-AR is the latest β-AR discovered. Although it was clear from its pharmacological profile that the β-AR family must contain a third member, the existence of β3-AR was only accepted when it was cloned, in 1989 [19]. β3-AR was finally recognized as a member of the β-AR family in 1994 [20]. Similar toother β-ARs, β3-AR is a member of the G protein-coupled receptor family and shares 50% sequence homology with β1-AR and 40% sequence homology with β2-AR. Unlike β1- and β2-ARs, β3-AR lacks phosphorylation sites in the third intracellular loop and C-terminal tail, which makes β3-ARs quite resistant to agonist-induced desensitization [21]. The gene coding for β3-AR (ADRB3) in humans is at position 8p11-12, distinct from those coding for β1- and β2-AR, and contains introns; therefore, it may undergo alternative splicing, generating different isoforms with pharmacologically distinct properties.

### 1.2. Animal Studies

Our interest in studying the role played by β-ARs began after the publication of a paper in 2008, which reported how the progression of infantile hemangiomas (IHs), a benign vascular tumor arising on a pre-existing hypoxic-ischemic lesion, could be effectively counteracted by the treatment with propranolol (a non-selective antagonist of β1- and β2-ARs) [22]. This clinical report paved the way to reveal the involvement of the adrenergic system (in particular of β2-AR) in neovascular processes mediated by hypoxia-induced VEGF upregulation, demonstrating for the first time that the close link between hypoxia and the triggering of vascularization could be decoupled by antagonizing β-ARs. At the same time, this finding promotes the exploration of the role of these receptors in other neonatal diseases, akin to the hypoxia-induced neovascularization seen with IHs, such as the retinopathy of prematurity (ROP) [23]. The studies carried out in an animal model of ROP have provided important and original contributions: on the one hand, the demonstration that propranolol was able to slow down retinal neoangiogenesis in this model allowed us to open a line of research (currently widespread in various countries), which is demonstrating the effectiveness of propranolol treatment in infants with ROP [24,25,26,27,28,29]. However, on the other hand, our preclinical studies evidenced that β3-AR is up-regulated during retinal vascular proliferation [30], a starting point that supported our characterization of its proangiogenic effect. Our research group has demonstrated, in fact, that this receptor is up-regulated in the retina during the development of hypoxia (with a trend completely comparable to that of HIF-1), and that it participates in the induction of VEGF production, and therefore in hypoxia-induced vascularization [31,32,33]. This is the reason why, while most research groups in the world were focused on evaluating the role of β1- and β2-ARs in a series of pathologies characterized by hypoxia-induced angiogenesis (including cancer), our group, taking advantage of the knowledge acquired through investigations on ROP, planned a series of studies to verify the potential involvement of β3-AR in cancer progression too [34,35,36,37].

In an article published in 2015, our research group analyzed some similarities between IHs, ROP, and cancer, three apparently very different diseases, but characterized by the common presence of hypoxia-induced angiogenesis. In all these pathologies, hypoxia, through the release of catecholamines, their interaction with β-ARs, and the activation of similar pathways, including the HIF-1/VEGF axis, was shown to be capable of inducing similar angiogenic processes. The presence of β3-AR in all these different scenarios, together with its hypoxia-induced up-regulation, allowed us to hypothesize a possible common pathogenic role of β3-AR in all these disorders and the existence of a proangiogenic HIF-1/β3-AR/VEGF axis [34]. Furthermore, in that article, we highlighted for the first time how this line of research had originated by studying either neonatal-onset diseases or animal models of fetal and neonatal diseases, in a scenario characterized by a hypoxic environment, a strong expression of β3-AR, and a modulation of vascularization by oxygen levels. We concluded the article with the observation that embryonic and fetal development in a physiologically hypoxic and catecholamine-rich environment (similar to the peritumoral environment) could suggest the fetal prototype as “an interesting model for understanding the mechanisms linking hypoxia, β-adrenergic system, and vascularization” [34].

Our speculation on analogies between cancer and embryos is only partially surprising, since it has been known for many years that cancer reactivates embryonic or fetal skills [38,39,40]. It is no coincidence that even today, many tumors are diagnosed through the presence of embryonic/fetal markers in the patient’s blood [41]. Following this idea, in recent years, we have demonstrated how the up-regulation of β3-AR induced by hypoxia could allow both embryonic and tumor cells to proliferate in a hypoxic environment [35,36,37], adapt their metabolism through the induction of the Warburg effect [42], acquire chemoresistance [43], and, finally, induce immune tolerance [44,45].

Some literature data suggest that the adrenergic system, and β3-AR in particular, may play an important role during embryonic and fetal life:

I. Catecholamines are crucial in the first weeks of intrauterine life, as demonstrated by the high embryonic lethality observed in mice lacking the expression of tyrosine hydroxylase [14,15,16];
II. β3-AR is expressed in the oocytes of various mammals [46,47] and in spermatozoa, in which it induces motility [48], thus promoting conception;
III. β3-AR is expressed in preimplantation embryos [47], during the early stages of embryogenesis [49], in embryonic tissues, and the placenta [50,51];
IV. Finally, β3-AR is up-regulated in the human pregnancy myometrium, where it inhibits spontaneous contractions and represents the predominant β-AR subtype [52,53], providing evidence that allows us to suggest β3-AR agonists as potential tocolytic drugs [54,55].

All these observations, combined with the fact that β3-AR expression is induced by a hypoxic environment, legitimized the idea that β3-AR is actively involved both in cancer progression and in the very early stages of life. Consequently, our idea was that many of the functions harmfully mediated by β3-AR in cancer are probably helpful during intrauterine life, and that the advantages for the embryo and fetus of dwelling in a hypoxic environment could be, at least in part, attributable to the high expression of β3-AR in various anatomical regions. Therefore, as a natural consequence of this reasoning, the idea that the common pathologies that develop in preterm newborns may be due, at least in part, to the lack of benefits that β3-AR would have brought in utero during the last weeks of pregnancy, began to take shape [56]. For this reason, our attention is now focused on the expression and the role of this receptor during embryonic and fetal life.

Some studies performed by our research group in various animal models demonstrated that the expression of β3-AR was not only modified by exposure to hypoxia but also by exposure to a hyperoxic environment. In the mouse, we have recently demonstrated that in the retina, the β3-AR is particularly expressed during intrauterine life, andthat its expression is reduced with birth and therefore with the passage from a relatively hypoxic environment to a relatively hyperoxic one [57]. In another recently published paper, we have demonstrated how in the mouse fetus β3-AR is expressed in the endothelial cells of the ductus arteriosus and participates in maintaining its patency. As soon as the mouse is born, and therefore exposed to a relatively hyperoxic environment, this receptor tends to down-regulate, thus favoring the closure of the duct [58]. In essence, the data available so far, obtained from animal models, suggest that β3-AR is actively modulated by oxygen levels, up-regulated in the intrauterine environment, and down-regulated in the extrauterine environment. This modulation of β3-AR expression as a function of different oxygen levels could explain why in the mouse fetus, β3-AR is highly expressed in the first days after conception and then, probably after placentation and therefore the increasing oxygenation, progressively tends to decrease [49].

If it is true that β3-AR plays an important role during the intrauterine life, favoring implantation, embryo growth, and, finally, fetal development and vascularization, then it is possible that premature birth deprives preterm newborns of the benefits that this receptor would have provided. This consideration is even more valid if we consider that exposure to a relatively hyperoxic environment, such as the extrauterine one, induces a down-regulation of β3-AR. These considerations have formed the basis of the attempt to limit the damage of early exposure to hyperoxia using β3-AR agonists [56]. In fact, the objective that we are currently pursuing is to evaluate whether treatment with a β3-AR agonist can decouple the exposure of some animals to hyperoxia from oxygen damage, primarily from vascular regression [56,59].

We recently published the first of a series of papers demonstrating how β3-AR agonism is able to significantly limit hyperoxia damage. In this study, we report how exposure to high concentrations of oxygen induces severe anatomical damage to the colon and a significant reduction in the number of colonic cells expressing β3-AR. Interestingly, the administration of a β3-AR agonist significantly prevented colonic length reduction, restored the production of mucins, and, surprisingly, reduced the alteration of the colonic myenteric plexus [60], suggesting that β3-AR agonism may represent a future therapeutic option for disorders induced by hyperoxia-impaired development, typical of prematurity disorders.

### 1.3. From Animals to Humans

While extensive information about the modulation and function of β3-AR in rodents, including its putative involvement in fetal well-being, is available, knowledge in humans is decidedly poorer. β3-AR reveals a restricted expression pattern in humans [61], but its involvement in the regulation of bladder smooth muscle tone suggests the employment of the β3-AR agonism in the treatment of overactive bladder syndrome in adults [62] and children [63].

β3-AR is expressed in myocardial tissue at a lower level if compared to the more abundant β1 and β2-AR [64], but β3-AR is up-regulated by hypoxia in failing hearts [65], where it participates in coronary vasodilation, through the intermediation of nitric oxide, and exerts negative inotropic and a positive lusitropic effects [66]. Its pharmacological agonism has recently been investigated in patients with left ventricular hypertrophy [67]. The presence of β3-AR in the human myometrium has been known for many years, as well as its increased expression during pregnancy [50,51,52,53,54,55]. However, so far, the interest in this receptor has been focused exclusively on its contractility-reducing effects and therefore as a potential target for tocolytic drugs.

Currently, our main objective is to obtain information regarding the expression of β3-AR during intrauterine life, with the aim of evaluating in humans whether its expression parallels oxygen levels, and whether its dynamic is in line with what was observed in the animal model. However, before being able to interpret any fluctuations in the expression of β3-AR, it was necessary to know with some accuracy how oxygen concentrations change during a normal pregnancy. Until recently, information on the placental and fetal oxygenation status was fragmentary and imprecise. It was known that the first stages of pregnancy were characterized by a very low level of oxygen, comparable to that of the non-pregnant uterus, even though the development of the placenta during the following weeks promotes a growing oxygen availability to the feto-placental unit that reaches a maximum at approximately 16 weeks of gestation [1,68]. From this point onwards, a gradual and progressive oxygen reduction has been reported both in the placenta and in fetuses [69,70].

In recent studies [71,72], evaluating a large series of umbilical cord blood gas analyses collected at birth from preterm and full-term neonates, we have demonstrated that intrauterine oxygen levels fluctuate throughout pregnancy, continuing to vary even after placenta development, thus showing a clear biphasic trend. Human fetuses, in fact, from mid-gestation (23rdweek) to near-term, become progressively more hypoxemic, despite the oxygen content remaining stable thanks to increasing hemoglobin concentration. However, starting from the 33rd–34thweek, oxygenation progressively increases until birth, probably as a consequence of the structural alterations of the aging placenta, which lead to an increase in oxygen diffusion [73]. Therefore, the placenta represents the hub that ensures this variable oxygen availability to the fetus, and we speculate that this biphasic trend is functional in promoting stemness and intrauterine differentiation, in specific tissues and at specific times [74].

### 1.4. Aim of the Present Study

The present paper aims to present a research protocol intended to virtually reconstruct the β3-AR expression level during fetal life and in human newborns during the first days of life.

The observation that in humans the expression levels of HIF-1, β3-AR, and VEGF show a trend similar to those observed in mice embryos, fetuses, and newborns would suggest the translatability of our preclinical observations to humans.

In particular, the investigation protocol will measure the expression of HIF-1, β3-AR, and VEGF in preterm and full-term newborns and correlate it with clinical data using multivariate analysis. Our working hypothesis is that premature birth deprives newborns of the benefits of prolonged exposure to a hypoxic environment, which in turn would have promoted the up-regulation of the HIF-1/β3-AR/VEGF axis. The earlier the premature birth, the less expressed the HIF-1/β3-AR/VEGF axis, and the worse the effects on fetal development, leading to more serious pathologies strictly related to prematurity.

We will include β1-AR and β2-AR expression measurement, to investigate whether any compensatory variation within the beta-adrenergic receptor family may occur.

## 2. Materials and Methods

### 2.1. Population

We designed a prospective, non-profit, single-center observational study. Our protocol has been approved by the Pediatric Ethical Committee for Clinical Research of the Tuscany region (number 291/2022).

Within 18 months, we will enroll 100 preterm newborns (group A, i.e., 23–37 weeks of gestational age) and 100 full-term newborns (group B, i.e., ≥37 weeks of gestational age) of any weight, of both genders, delivered via vaginal delivery or cesarean section at Pisa University Hospital (Italy). Written consent will be obtained from the parents of eligible infants, after being adequately informed about the trial either by the principal investigator or by the collaborators. Denied consent will be the only exclusion criteria.

In group A, the following samplings will be performed (0.5 mL each):

▪ Cord blood sampling (T0);
▪ Capillary blood sampling at 48–72 h (T1);
▪ Capillary blood sampling at 14 days (T2);
▪ Capillary blood sampling at 30 days (T3);
▪ Capillary blood sampling at 40 ± 3 weeks of post-menstrual age (T4), regardless of days of life.

**Figure 1:**
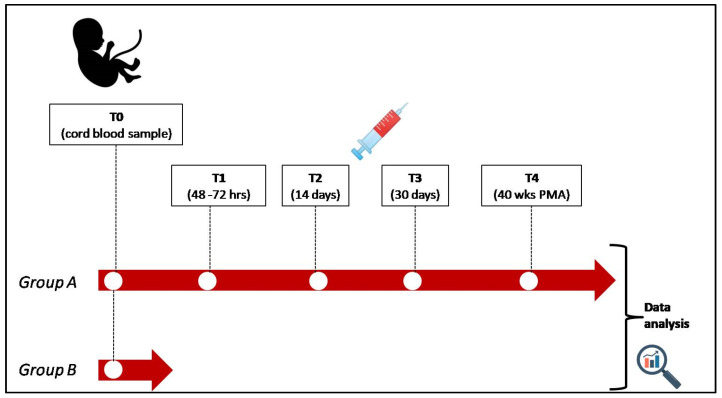
Research protocol timeline (group A: preterm newborns, group B: term newborns; PMA: post-menstrual age).

In group B, only a cord blood sampling (T0) will be collected (Figure 1).

For cord blood sample collection, an approximate 20 cm long segment of the cord will be isolated and cut between a set of two clamps. Cord blood will be collected by a syringe and then put in EDTA-tube.

For group A, clinical data relating to the main causes and complications of preterm birth will be collected, including the following:

▪ The presence of infarct areas, or evidence of chorioamnionitis, or pregnancies identified through the pathological study of the placenta;
▪ Apgar score at 5′ and 10′;
▪ Resuscitation at birth;
▪ Need for oxygen therapy (duration, mode, maximum FiO2);
▪ Diagnosis of bronchopulmonary dysplasia (BPD);
▪ Diagnosis of patent ductus arteriosus (PDA) and related treatment (pharmacological/surgical, outcome);
▪ Diagnosis of necrotizing enterocolitis (NEC);
▪ Diagnosis of ROP (stage, treatment methods, outcome);
▪ Diagnosis of periventricular leucomalacia (PVL).

Furthermore, for both group A and group B patients, the following information will also be collected, to be considered during the final data analysis: gestational age, ethnicity, gravidity, parity, gender, birth weight, the birth of small for gestational age (SGA) infants, or infants with signs of intrauterine growth restriction (IUGR), maternal gestational diabetes, pre-pregnancy maternal diabetes, maternal diabetes therapy, maternal preeclampsia/eclampsia, mode of delivery (spontaneous/cesarean), maternal celiac disease, intake/abuse of substances or drugs, prenatal prophylaxis with steroids.

### 2.2. Blood Sample Processing and RNA Isolation

Blood samples will be frozen at −80 °C until use (i.e., RNA isolation and analysis of ADRB3, HIF, and VEGFA expression).

RNA will be isolated from 500 µL of blood using the QIAamp RNA Blood Mini kit (Qiagen, Valencia, CA, USA), according to the manual instructions.

The isolation procedures provide a rapid and simple method for the extraction of total cellular RNA without the use of toxic substances such as phenol and/or chloroform. Instead, they are replaced by the QIAamp procedure, i.e., columns with high-affinity membranes. This method ensures the complete removal of contaminants and enzyme inhibitors, such as hemoglobin and heparin, using a selective silica-based membrane (QIAamp membrane—spin column). In detail, erythrocytes are selectively lysed, and leukocytes are recovered through centrifugation. Subsequently, the leukocytes undergo lysis under highly denaturing conditions, promptly deactivating RNases and facilitating RNA isolation. Following the homogenization of the lysate through brief centrifugation with the QIAshredder spin column, ethanol is used to adjust the binding conditions. The resulting mixture is then applied to the QIAamp spin column, where RNA binds to the silica membrane during a brief centrifugation. Contaminants are washed away, and total RNA is eluted in 35 μL of RNase-free water.

The end result is purified RNA, ready for use in various downstream applications, including gene expression analysis via digital PCR (dPCR).

### 2.3. Measurement of ADRB3, HIF, and VEGFA RNA Levels Using Digital PCR

The RNA levels of ADRB3, HIF, and VEGFA will be measured using the QIAcuity Digital PCR System (Qiagen, Valencia, CA, USA), the QIAcuity^®^OneStep Advanced Probe Kit (Qiagen, Valencia, CA, USA), and the PrimePCRddPCR Gene Expression Probe Assay (Bio-Rad, Hercules, CA, USA) for ADRB3 (human), HIF-1 (human), and VEGFA (human). The human β-actin (ACTB) ddPCR assay (Bio-Rad, Hercules, CA, USA) will be used as a reference gene and internal control.

PCR reactions will be assembled into individual wells (96-well PCR plate) according to the following protocol: 4 μL of RNA, 10 μL 4× OneStep Advanced Probe Master Mix (Qiagen), 0.4 μL 100× OneStep Advanced RT Mix (Qiagen), 1 μL 20× primer/probe assay (target gene—FAM), 1 μL 20× primer/probe assay (reference gene—HEX), 23.6 μL RNase-free water (total volume: 40 μL).

The solution will then be transferred into the wells of a 26k 24-well nanoplate (Qiagen, Valencia, CA, USA) and sealed using the QIAcuity Nanoplate Seal provided in the QIAcuity Nanoplate Kit (Qiagen, Valencia, CA, USA). The following conditions will be used for the reverse transcriptase PCR reaction: 50 °C × 40 min, 95 °C × 2 min, 95 °C × 5 s, and 55 °C × 30 s (40 cycles). After PCR, the amplification target will be acquired by measuring the fluorescence in all positive partitions. The QIAcuity software suite will analyze the number of positive versus negative partitions for both fluorophores (FAM/HEX), to calculate the average amount of target RNA using the Poisson distribution. In the present study, the expressions of our target genes will be normalized to the expression of the reference gene (ACTB). Data will be reported as ratio (%), calculated as follows: absolute quantification of mRNA copies of each biomarker (copies/mL)/absolute quantification of mRNA copies (copies/mL) of β-actin (ACTB) as a housekeeping gene. The use of an internal control allows us to exclude bias in leucocytes enumerations.

### 2.4. Statistics

Finally, we will carry out the appropriate statistical analysis to compare the results obtained in group A (T0) vs group B (T0), and to identify any correlations between the values obtained from the serial samples in group A and the clinical data of the patients. To determine the reference interval of the RNA levels for the expression of the 3 studied genes measured in the blood, we will construct the relevant curve with a 95% confidence interval using polynomial regression. To compare the means of independent groups, we will use the *t*-test for independent samples. To study the relationships between the quantitative variables, we will calculate the Pearson correlation coefficient. For the global analysis of outcome predictability, we will carry out a multivariable logistic regression.

According to recent recommendations on sensitive data management and patients’ privacy, an appropriate electronic password-protected access system for the correct de-identification/anonymization, collection, and management of patients’ data will be used. Participants’ identifying information will be stored separately, with limited access.

## 3. Discussion

### 3.1. Expected Results

**Figure 2:**
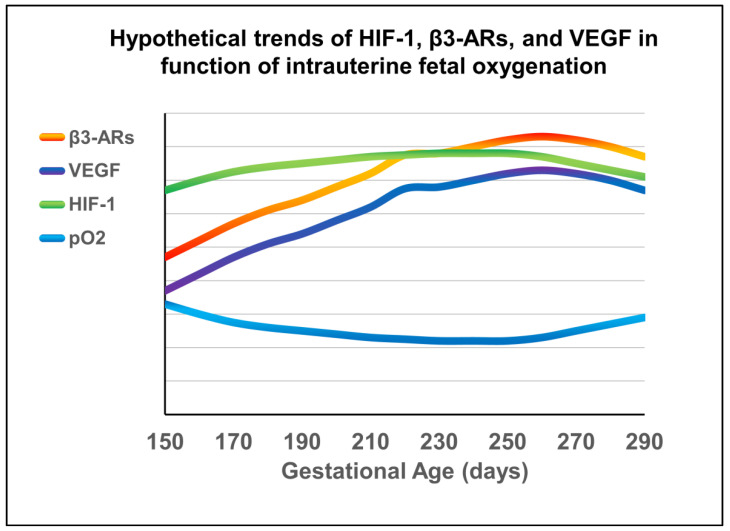
Based on previous measurements of intrauterine oxygen fluctuations (blue line) in human newborns, we imagine a hypothetical trend of HIF-1 (green), β3-AR (orange),and VEGF (purple) expression. The research protocol detailed in this paper is aimed at verifying our hypothesis.

**Figure 3:**
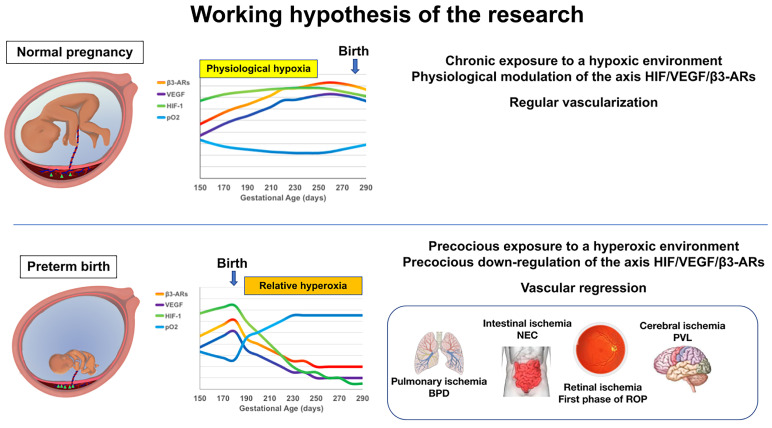
Our research protocol intends to verify the hypothesis about the expression trend of the HIF-1/VEGF/β3-AR axis. If it corresponds to reality, then we can also speculate that preterm birth and the consequent exposure to hyperoxia may cause down-regulation of these genes and therefore a vascularization arrest (or regression) in critical organs.

Our working hypothesis is that the birth of a preterm newborn induces the arrest of fetal vascularization (which is still immature) through a down-regulation of the HIF-1/β3-AR/VEGF axis, similar to what has been recently observed in animals (Figure 2 and Figure 3).

Therefore, as primary aims, we expect to accomplish the following tasks:

I. Verify whether the expression of β3-AR in preterm newborns differs from that in full-term newborns and whether this expression varies as a function of the severity of prematurity;
II. Verify whether the expression of HIF-1, β3-AR, and VEGF during intrauterine life differs from that observed after birth;
III. Verify whether the postnatal expression of the HIF-1, β3-AR, and VEGF is modulated by oxygen exposure.

Moreover, as secondary aims, we will try to accomplish the following tasks:

I. Correlate the expression levels of HIF-1, β3-AR, and VEGF with the onset of the main complications of preterm birth (ROP, BPD, PVL, NEC);
II. Study possible changes in β3-AR expression levels in case of peripartum hypoxic events.

### 3.2. Discussion

It has been known for decades that preterm birth implies a not-yet-completed vascularization, especially in organs such as the retina, lungs, central nervous system, and intestine [75,76,77,78], and that the main pathologies of the premature newborn (the first phase of ROP, BPD, PVL, NEC) display vascular regression, induced by early exposure to oxygen as their initial triggering event [56].

In light of the recent evidence that attributes a key role to β3-AR in the coupling between hypoxia and vascularization [31,56,59], the main aim of the present project is to evaluate the expression level of β3-AR during fetal life and in human preterm newborns after their exposure to a relatively hyperoxic environment. A significant advantage of the present investigation is that it is an absolutely non-invasive methodology. Indeed, cord blood sampling does not cause pain for either the newborn or the mother. After that, we will collect blood samples in preterm newborns (T1, T2, T3, T4) only during routine medical care, when other blood tests are being carried out.

During fetal life, it is possible that different organs have variable exposure to hypoxia, and therefore the expression of β3-ARs might vary among tissues. Indisputably, blood cells are among the cells least exposed to hypoxia during intrauterine life. However, the use of this tissue is the only strategy to perform gene expression studies on fetal cells obtained in a non-invasive and painless manner. In any case, if we observe a modification in the expression of the HIF/VEGF/β3-ARs axis in cells (such as blood cells) that are exposed to a modest change in oxygenation, in tissues exposed to deeper hypoxia, the modulation of the HIF/VEGF/β3-ARs axis is likely to be even greater. For this reason, in our opinion, blood cells can be a useful indicator of the expression levels of the HIF/VEGF/β3-ARs axis in relation to oxygen levels.

Particular attention will be paid to evaluating whether during intrauterine life in humans, the expression of β3-AR reflects the trend of oxygen levels and parallels that of HIF-1, as previously observed in preclinical models. In the mouse retina, where it is well known that the expression of HIF-1 and β3-AR is up-regulated by hypoxia, our research group recently demonstrated that HIF-1 acts as a transcription factor involved in the regulation of the ADRB3expression [79]. In this context, it is very likely that in humans as well, HIF-1 directly modulates β3-AR transcriptional regulation. In fact, the examination of promoter regions of the human genes using PROMO [80], based on the TRANSFAC database [81] demonstrates that the human ADRB3 gene has a canonical HRE [82].

Therefore, we imagine that the fetus shows a coordinated fluctuation of the different proangiogenic factors stimulated by fluctuating oxygen levels; for this reason, we imagine that the biphasic trend of pO2 that we have recently reconstructed through the analysis of umbilical blood samplings [71,72] induces in turn a biphasic expression of HIF-1, β3-AR, and VEGF. Figure 2 represents the kinetics of HIF-1, β3-AR, and VEGF that we expect to find by analyzing umbilical blood samplings.

We are aware that our study has limitations. In particular, it is a pilot study, i.e., hypothesis-generating work. For this reason, we will explore a set of data in order to verify if preclinical findings can be translated to human beings. Then, we will hopefully be able to extend measurements to other tissues (first of all, the placenta) and also include protein detection with their cellular localization using flow cytometry. Finally, we will be hopefully able to propose hypotheses which may be tested in some subsequent studies, eventually leading to case-control studies.

### 3.3. Conclusions

If we better understand the mechanisms underlying the well-being of embryos and fetuses in the intrauterine environment, i.e., in conditions characterized by physiologically low levels of oxygen, it certainly becomes easier to understand why premature newborns suffer so much from early exposure to a relatively hyperoxic environment. Consequently, this awareness could better allow us to face the main pathologies related to prematurity. Indeed, if the results of the study are in line with our predictions, and therefore it will be demonstrated that in humans the behavior of β3-AR is similar to that observed in rodent models, this would open up a new therapeutic scenario for the prevention of the main pathologies related to prematurity, which could theoretically benefit from the pharmacological agonism of β3-ARs after birth [56].

## Data Availability

Not Applicable.

## Abbreviations

HIF-1	Hypoxia-Inducible Factor-1
VEGF	Vascular Endothelial Growth Factor
β3-AR	β3-adrenoreceptor
HREs	hypoxia-responsive elements
IHs	infantile hemangiomas
BPD	bronchopulmonary dysplasia
PDA	patent ductus arteriosus
NEC	necrotizing enterocolitis
ROP	retinopathy of prematurity
PVL	periventricular leucomalacia
SGA	small for gestational age
IUGR	intrauterine growth restriction
